# Hygiene management for long-term ventilated persons in the home health care setting: a scoping review

**DOI:** 10.1186/s12913-022-07643-w

**Published:** 2022-02-23

**Authors:** Isabel Hoeppchen, Carola Walter, Stefanie Berger, Anna Brandauer, Nicole Freywald, Patrick Kutschar, Katharina Maria Lex, Annemarie Strobl, Irmela Gnass

**Affiliations:** Institute of Nursing Science and Practice, Paracelsus Medical University Salzburg, Strubergasse 21, Salzburg, Austria

**Keywords:** Artificial ventilation, Shared living community, Home mechanical ventilation, MRSA, Outpatient care, Infection prevention, Infection control, Evidence-based practice, Nursing service, Quality management

## Abstract

**Background:**

Evidence and recommendations for hygiene management in home mechanical ventilation (HMV) are rare. In Germany, few regionally limited studies show poor hygiene management or a lack of its implementation. This scoping review of international literature identified the evidence in hygiene management for ventilated patients in the home care setting which has to be implemented for infection prevention and control.

**Methods:**

A review of international literature was conducted in CINAHL, PubMed and Web of Science. The search focused on four key domains: HMV, hygiene management, home care setting, and methicillin-resistant Staphylococcus aureus (MRSA). Data of included studies were extracted using a data charting sheet. Extracted data were assigned to the categories (1) study description, (2) setting and participants, and (3) hygiene management.

**Results:**

From 1,718 reviewed articles, *n* = 8 studies met inclusion criteria. All included studies had a quantitative study design. The approaches were heterogeneous due to different settings, study populations and types of ventilation performed. Regarding aspects of hygiene management, most evidence was found for infectious critical activities (*n* = 5), quality management for hygiene (*n* = 4), and training and education (*n* = 4). This review identified research gaps concerning kitchen hygiene, relatives and visitors of HMV patients, and waste management (*n* = 0).

**Discussion:**

Overall evidence was rather scarce. Consequently, this review could not answer all underlying research questions. No evidence was found for measures in hygiene management relating to ventilated patients’ relatives. Evidence for kitchen hygiene, waste management and interaction with relatives is available for inpatient care settings. However, this may not be transferable to outpatient care. Binding legal requirements and audits may help regulate the implementation of HMV hygiene measures.

**Conclusion:**

Infection control programmes included qualified personnel, hygiene plans, and standards for MRSA and multidrug-resistant organisms (MDRO). The appropriateness of hygiene management measures for outpatient care is the basis for their application in practice.

**Supplementary Information:**

The online version contains supplementary material available at 10.1186/s12913-022-07643-w.

## Background

Home mechanical ventilation (HMV) is an established treatment for patients with chronic respiratory failure. HMV is defined as non-invasive ventilation via a mask or invasive ventilation via tracheostomy [1]. According to the Association of Scientific Medical Societies (AWMF), the ventilation is applied in the user’s home or other long-term care facilities, usually not in hospital settings [2]. In Germany, HMV patients also can live in shared living communities (SLC). SLC are defined as communities with a maximum of twelve persons who live in rented facilities or apartments [3]. Care is ensured through an optional outpatient care service by qualified nursing staff [3]. The frequency of SLC varies between the federal states and is reported to range from 0 (Saarland) to 690 (Berlin) [3]. It is estimated that 12% of all SLC nationwide are intensive care SLC [3].

According to the current state of research, the prevalence for HMV varies widely: While Valko et al. [4] estimated the prevalence for Hungary at 3.9/100,000 in 2018, Vitacca et al. [5] estimated a prevalence of 63/100,000 in Italy in 2012. A Europe-wide study, which included 16 countries, mentioned a prevalence of 6.6/100,000 in 2005 [1]. It is estimated that the number of patients requiring HMV is increasing internationally [6–8]. In Germany, however, there is limited data eligibility on HMV or long-term mechanical ventilation in general [9].

Due to artificial respiration, patients with HMV require intensive care. Ventilation is associated with the frequent use of medical devices, such as tracheal cannulas, catheters, and gastric tubes [10]. In addition, a high hospitalisation rate, co-morbidities, and a significantly increased probability of respiratory tract infections represent risk factors for colonisation with methicillin-resistant Staphylococcus aureus (MRSA) [11]. A study on MRSA-colonisation of ventilated patients living in SLC estimated a prevalence of 29.6% [10]. Therefore, adherence to recommendations of hygiene standards published by the *Robert-Koch Institute* (RKI) [12] is necessary especially, to prevent respiratory tract infections, which are considered the main reason for hospitalisation of home care patients [13].

So far, there are only a few regionally limited studies on hygiene management in outpatient intensive care in Germany [14]. There is evidence in the literature that proper hygiene management and prevention strategies are either not well-known or not consistently implemented in practice [10, 15, 16]. Among other factors, these findings are attributed to a lack of regulatory support and also demonstrate the need for binding legal guidelines. However, these challenges are not specific to the German health care setting. It is also known from international literature that there are differences in infection control policies and practices, especially in outpatient care [13, 17, 18].

In Germany, the monitoring of hygiene management in outpatient care is carried out by different control authorities in each federal state [3]. In Bavaria, for example, the monitoring of care services is executed by the *Department of Health and Environment* (RGU) and the *Medical Service of Health Insurance* (MDK) [10]. To address the need for standard procedures and binding guidelines, the German government approved an amendment to the *Law to Strengthen Intensive Care and Medical Rehabilitation* (IPReG) in October 2020 [19]. In addition, the *Society for Outpatient Intensive Care* (KNAIB) [20] published hygiene standards for outpatient care, which describes requirements for structural and process quality. In contrast to inpatient care, current literature lacks summarizing evidence and recommendations for hygiene measures in outpatient care, including SLC.

### Aims

To address the existing challenges about hygiene management in Germany, this review aimed to identify the evidence in hygiene management for ventilated persons in the home care setting, which has to be implemented for infection prevention and control. While these findings are particularly relevant to the German context, they may also be applicable in other countries facing similar problems. A scoping review of international literature was conducted concerning the following primary research question: Which measures of hygiene management are recommended for long-term invasively and non-invasively ventilated persons in the home care setting?

Target group-specific research questions were formulated to present evidence appropriately:

1. Which measures of hygiene management are recommended for health care professionals in the home care setting?
2. Which measures of hygiene management are recommended for ventilated persons in the home care setting?
3. Which specific measures are mentioned for hygiene management for persons either infected or colonised with MRSA?
4. Which measures of hygiene management are recommended for relatives of ventilated persons in the home care setting?

## Methods

This scoping review reports in accordance with the Preferred Reporting Items for Systematic Reviews (PRISMA) extended for Scoping Reviews by Tricco et al. [21]. Moreover, the review was based on the methodological framework for scoping review principles defined by Arksey and O’Malley [22]. The research protocol was registered with the Open Science Framework (https://doi.org/10.17605/OSF.IO/TZG8H).

### Search strategy

A search of English and German literature was performed up until 21.07.2020. Three databases were searched (AS, CW, IG): CINAHL, PubMed and Web of Science. Manual search was also carried out in Google Scholar. Searching for additional sources was completed by 31.10.2020.

The search strategy for the databases was derived from the research questions and related to four key domains: HMV, hygiene management, home care setting, and MRSA. The search terms were developed for the defined search domains and then adapted to the three databases according to RefHunter, Version 4.0 [23]. The comprehensive search strategy is exemplified for CINAHL in Appendix 1.

### Eligibility Criteria

As inclusion and exclusion criteria, characteristics regarding settings, patients, research topics, study designs, and publication types were used. Due to the focus of the research project on home mechanical ventilation in adults, studies with participants under 18 years of age were excluded. Two authors (AS, CW) screened all titles and abstracts for eligibility. The remaining full-texts were assessed against different inclusion and exclusion criteria (CW, IG), namely setting-, patient-, or study-related (Tab. 1). In case of discrepancies, a third author (PK) was involved to reach a consensus.

**Table 1:** Definition of inclusion and exclusion criteria

	Inclusion Criteria	Exclusion Criteria
Setting-based criteria	•Outpatient •Home care setting (e.g. SLC, skilled nursing facilities, long-term care facilities)	•Intensive Care Unit •Hospital •Rehabilitation
Patient-based criteria	•Age ≥ 18	•Age < 18
Topic-based/ phenomena-related criteria	•HMV •Hygiene management	•Studies with focus on changes of pulmonary functions •Studies with primary focus pharmacological testing, e.g. antibiotics
Study design criteria	•Qualitative research •Quantitative research •Mixed methods research	
Publication type criteria	•Language: German and English	•Abstracts, Letters, Editorials •Reviews •Development of instruments •Medical case studies •Expert opinion •Policy/ legal documents

### Data extraction and charting

A data charting sheet was developed by CW and IG (Appendix 2 ). The data charting sheet was piloted on a randomly selected paper and used for the remaining studies. Eligible sources were reviewed and extracted by IH. All extracted data were checked by CW and IG. Find the data charting sheets for all included studies in Appendix 3 .

The key findings from the studies were assigned to the following categories of the data charting sheet: (1) the study description: first author, study title, year of publication, country, study aims, methodology/measures, (2) the setting and participants: sample size, sex, participants’ age, kind of disease, kind of artificial ventilation, family participation, concept of home-based setting, professions, cooperation. The categories used for extracting data on (3) hygiene management based on the checklist for hygiene management in outpatient intensive care reported by KNAIB in 2019 [20]: *Quality Management for Hygiene*, *Training and Education*, *Staff Hygiene*, *Relatives and Visitors*, *Cleaning and Disinfection Aspects*, *Handling of Medical Devices*, *Waste Management*, *Infectious Critical Activities*, *Caring for Infected Persons*, *Handling of Medication*, *Laundry Hygiene*, and *Kitchen Hygiene*.

The extracted data were summarized systematically: First, tables and figures were designed to display extracted data. Secondly, data were analysed in line with the research questions and synthesised narratively.

Since the central purpose of scoping reviews is to reflect the extent, the characteristics and the variance in the literature regarding the research topic [21], a critical appraisal and evaluation of the methodological quality was not performed.

## Results

### Literature search

**Fig. 1:**
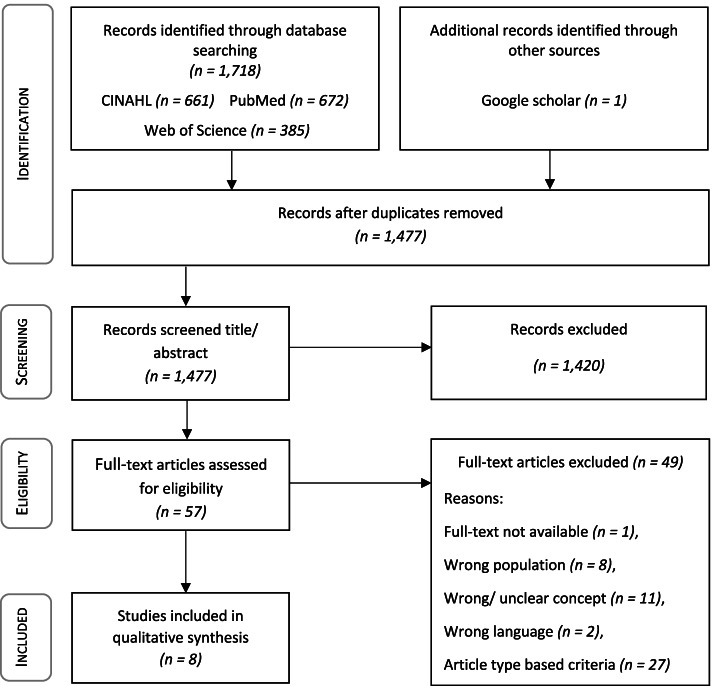
PRISMA flow chart of the study selection process

The electronic database search identified 1,718 articles. One additional record was identified via Google Scholar. In the first exclusion phase, duplicates were removed leaving 1,477 sources for the title and abstract screening. All in all, 57 articles were potentially relevant, and full-texts were reviewed for eligibility along with the inclusion and exclusion criteria. Eight articles were identified for inclusion in this scoping review and remained for qualitative synthesis. The PRISMA flowchart (Fig. 1) describes the study selection process.

### Characteristics of included studies

The included studies were published between 1997 and 2020 (s. Tab 2). All studies had a quantitative study design, whereby four studies [24–27] analysed microbiological swabs taken from patients or medical devices. Five studies were conducted and published in Europe (Germany, Belgium and France) [24, 25, 27–29], and three studies were carried out in the USA [26, 30, 31].

**Table 2:** Characteristics of included studies

Authors, publication year	Country	Studies’ Settings	Home Care Setting	Objectives	Study design	Data collection/ analysis
Cahill et al., 1997 [31]	USA	SNF (hospital-based distinct-part and free-standing)	SNF	**(1)** Characteristics of infection prevention and control program, **(2)** policies for admitting and placing of MRSA- or VRE-colonized patients, **(3)** educational needs for infection prevention and control programs in skilled nursing facilities	Descriptive Study	Quantitative survey among ICPs
Toussaint et al., 2006 [24]	Belgium	Centre for Home Mechanical Ventilation, Brusseles	Patients’ home	**(1)** HVC cleanliness and sterility of HVC, **(2)** efficiency of tubing cleaning and decontamination protocols recommended to patients	Non-randomised controlled trial	39 used and 7 new EVAs (as control) were examined in 2 different experiments (visual and microbiological analysis)
Banfi et al., 2007 [29]	France	At patients’ home	Patients’ home	Effectiveness and safety of home care treatment of ARF	Non-randomised controlled trial	Daily duration of ventilation and antibiotics (supervision of GP, nurse, and chest specialist)
Chenoweth et al., 2007 [30]	USA	University-affiliated Home Care Services department, Michigan	Patients’ home	Characterise VAP in HMV patients: **(1)** rate and incidence, **(2)** demographic characteristics, **(3)** risk factors, **(4)** outcomes	Descriptive study	Data extraction from medical records (demographic and clinical variables)
Neumann et al., 2016 [25]	Germany	Nursing services (intensive care and normal care), Rhine-Main area	*Not specified*	**(1)** Prevalence of MDRO and risk factors for MDRO, **(2)** MDRO and MRSA-colonisation in intensive care services vs. other nursing services	Descriptive study	**(1)** Anamnestic survey in nursing homes, **(2)** microbiological analysis of throat, nasal and anal swabs
Prasad et al., 2016 [26]	USA	Long-term care facility in New York City	Long-term care facility	Prevalence of asymptomatic rectal colonisation with CRE or CDI in long-term care patients	Descriptive study	**(1)** Microbiological analysis of rectal swabs, **(2)** retrospective chart review for patient demographics and risk factors
Horvath et al., 2018 [28]	Germany	18 intensive care SLC in Munich	SLC	**(1)** type of care provided in outpatient intensive care SLC in Munich, **(2)** grade of implementation of hygiene and emergency management	Descriptive study	**(1)** Structure analysis, **(2)** inspection of SLC, **(3)** review report
Schwerdtner et al., 2020 [27]	Germany	3 intensive care SLC in Jena	SLC	**(1)** prevalence of MDRO in SLC, **(2)** evaluation of hygiene management in SLC	Descriptive study	**(1)** Occasional inspection on hygiene management, **(2)** microbiological analysis of devices and throat/ nasal and anal swabs, **(3)** structural analysis, anamnestic data on MDRO-colonisation

*ARF,* acute respiratory failure*,CRE* carbapenem-resistant enterobacteriaceae*,CDI* clostridium difficile*,EVA* expiratory valve*,GP* general practitioner,HVC home ventilation circuit*,ICP* infection control practitioner*,VAP* ventilator associated pneumonia*, MDRO* multidrug-resistant organisms*,SLC* shared living community*,SNF* skilled nursing facility*, VRE* vancomycin-resistant enterococci

The interventions were conducted in different settings, and patients included in the studies lived in various home care settings, e.g. SLC [27], skilled nursing facilities (SNF) [28, 31] or long-term care facilities [26]. Neumann et al. [25] did not describe the home care setting specifically. Regarding cooperation with other medical institutions, Horvath et al. [28] indicated that two of 18 SLC were affiliated with a weaning centre. Prasad et al. [26] mentioned cooperation with general practitioners and chest specialists to supervise patients with respiratory infections at home.

Three studies provided information regarding professions and qualifications. Cahill et al. [31] characterised infection control practitioners (ICPs) in SNF. They were qualified by having an associate degree or higher in “nursing education”, but seldom had a bachelor’s degree in nursing. Most of the ICPs were responsible for at least another non-infection control-related position. More than 50% of ICPs had a working experience of at least five years. Horvath et al. [28] described the qualification of leading nurses in nursing services and stated that most of them completed further training for intensive care, and one leader did a course on HMV. Schwerdtner et al. [27] found in total 60 employees in three SLC. Mostly, nurses were medical or geriatric nurses. Table 2 shows the characteristics of the included studies.

### Study Population

The sample sizes varied widely. For example, while Neumann et al. [25] included 486 patients from different nursing services, Banfi et al. [29] examined only eight patients. Regarding the studies’ population characteristics, the way of describing age and gender distribution differed between the included studies. For example, Neumann et al. [25] only reported the percentage of participants older than 85 years. In four studies [24, 27, 28, 31], data on age, sex, diseases and application of devices were missing. Table 3 shows sample sizes and characteristics of the studies’ population.

**Table 3:** Studies' population characteristics

Author	Sample size	Age	Sex	Diseases, comorbid conditions and devices
Cahill et al., 1997 [31]	444 SNF *(Number of patients not specified)*	*Not specified*	*Not specified*	*Not specified*
Toussaint et al., 2006 [24]	HVC of 39 patients	*Not specified*	*Not specified*	Respiratory, neurologic (functional tetraplegia with chronic alveolar hypoventilation)
Banfi et al., 2007 [29]	8 patients	61	63% male	ARF; comorbid conditions: respiratory (idiopathic severe kyphoscoliosis)
Chenoweth et al., 2007 [30]	57 patients	With VAP: 19 Without VAP: 14	With VAP: 48% male Without VAP: 43% male	VAP; comorbid conditions: respiratory, cardiovascular, gastrointestinal, diabetes mellitus, renal
Neumann et al., 2016 [25]	486 patients (normal nursing service n = 466; intensive care service n = 20)	Normal nursing service 37% (intensive care service 0%) > 85	Normal nursing service (intensive care service): 33% (50%) male	Respiratory, gastrointestinal, skin barrier violations, renal, orthopaedic, cognitive impairment
Prasad et al., 2016 [26]	301 patients	75	63% female	Respiratory, cardiovascular, diabetes mellitus, cognitive impairment
Horvath et al., 2018 [28]	85 patients living in 18 intensive care SLC	*Not specified*	*Not specified*	*Not specified*
Schwerdtner et al., 2020 [27]	24 patients	13% < 18	*Not specified*	Respiratory, gastrointestinal, renal, skin barrier violations In total 75 devices for 24 patients

*ARF* *acute respiratory failure, HVC*  *home ventilation circuits, SNF* *skilled nursing facility, VAP* *ventilator associated pneumonia*

Six included studies [24–27, 29, 30] described the type of ventilation used on the patients. Table 4 shows the percentages of invasively and non-invasively ventilated patients and information on the duration of ventilation.

**Table 4:** Types of ventilation

Author	Invasively ventilated	Non-invasively ventilated	Ventilation duration
Toussaint et al., 2006 [24]	41% (per tracheostoma)	59% (per nasal mask)	All patients ventilated at home with EVA for > 12 months (mean time ventilated: 7.7 years)
Banfi et al., 2007 [29]	12.5%	87.5% (per NPPV and nasal mask) 57.2% pressure assist ventilator and EVA 42.8% volume-assist ventilator	All patients had mechanical ventilation for a mean of 31 months
Chenoweth et al., 2007 [30]	100%	-	Mean duration of ventilation per patient: 890.6 days
Neumann et al., 2016 [25]	Intensive care service: 70% (per tracheostoma) Normal care service: 0%	*Not specified*	*Not specified*
Schwerdtner et al., 2020 [27]	88% per tracheostomy, but not all ventilated at time of study	*Not specified*	Ventilation time per day: 16.7% permanently 29.2% < 24 h
Prasad et al., 2016 [26]	41% had airway ventilation (not further described)		*Not specified*
Cahill et al., 1997 [31]	*Not specified*	*Not specified*	*Not specified*
Horvath et al., 2018 [28]	*Not specified*	*Not specified*	*Not specified*

*EVA*  *expiratory valve; NPPV*  *non-invasive positive pressure ventilation*

### Hygiene management

*Infectious Critical Activities* were addressed in five of eight included studies [25–27, 30, 31] (s. Tab. 5). Two studies examined risk factors for colonisation with multi-drug resistant pathogens (MDRO) [25, 26]. For example, Neumann et al. [25] found that a level of care dependency above three and high hospitalisation rates increase the risk of a MRSA colonisation. Two studies described the screening of new admissions for MDRO [27, 31]. Cahill et al. [31] stated that approximately 90% of ICP respondents reported not to screen new admissions for MDRO in SNF. Similar results were found by Schwerdtner et al. [27] in a different setting: Only one SLC reported screening new admissions. Furthermore, annual routine swabs are taken by a general practitioner, however, these were not performed systematically [27]. Regarding nursing activities, suctioning of secretions by using the clean technique in the treatment of ventilator associated pneumonia (VAP) in HMV patients was reported by Chenoweth et al. [30].

**Table 5:** Addressed aspects of hygiene management according to KNAIB [20]

Aspects of Hygiene Management												
	Infectious Critical Activities	Quality Management for Hygiene	Training and Education	Cleaning and Disinfection Aspects	Handling of Medical Devices	Handling of Medi-cation	Caring for Infected Persons	Staff Hygiene	Laundry Hygiene	Kitchen Hygiene	Relatives and Visitors	Waste Manage-ment
Cahill et al., 1997 [31]	x	x	x				x					
Toussaint et al., 2006 [24]			x	x	x							
Banfi et al., 2007 [29]						x						
Chenoweth et al., 2007 [30]	x				x							
Neumann et al., 2016 [25]	x			x		x						
Prasad et al., 2016 [26]	x	x										
Horvath et al., 2018 [28]		x	x	x	x		x	x	x			
Schwerdtner et al., 2020 [27]	x	x	x			x		x				

Four included studies addressed *Training and Education* [24, 27, 28, 31]. Two studies described that regular hygiene education courses are held in SLC for nursing staff [27, 28]. Based on their findings, Schwerdtner et al. [27] concluded there is a need for training regarding preparation of medical devices and basis hygiene aspects among nursing staff in SLC. Cahill et al. [31] through a survey of ICPs in SNF on educational needs found that the most relevant topics were about hand washing, pathogens’ standards, MRSA, and appropriate use of antibiotics. Regarding education for patients, Toussaint et al. [24] found that patients rarely adhered to maintenance advice protocols, even if it was taught and handed out in written form.

*Quality Management for Hygiene* was addressed in four included studies [26–28, 31]. Presence of isolation practice was described by Prasad et al. [26] and Cahill et al. [31]. Also, two studies described the presence of ICPs or hygiene representatives in the respective care setting [28, 31]. Cahill et al. [31] further described an obligatory hygiene management programme in SNF and the average time for infection prevention and control activities. Schwerdtner et al. [27] assessed the hygiene management of an SLC in Jena as deficient with missing structural and technical requirements of the building. Regarding hygiene plans, Horvath et al. [28] found them available in all studied SLC. However in some cases, employees did not have access to hygiene plans. Furthermore, standards for MDRO were partly missing [28]. Nearly all of the inspected SLC had standards for endotracheal suctioning and handling of tracheal cannulas [28].

*Cleaning and Disinfection Aspects* were mentioned in three included studies [24, 25, 28]. Toussaint et al. [24] compared different preparation methods for whole HMV circuits and recommended a low-level disinfection of all pieces of the circuit in the dishwasher (90 min at 70°). Horvath et al. [28] reported the presence of certified disinfectants for surface and hand disinfection in SLC. Another study reported that sanitary kits were made available in case of MRSA-detection during an inspection of an SLC [25].

Three included studies addressed *Handling of Medical Devices* [24, 28, 30]. Horvath et al. [28] stated that in some inspected SLC in Munich preparation of tracheal cannula was not performed properly. Toussaint et al. [24] studied a variety of cleaning procedures for HMV circuits. Chenoweth et al. [30] reported a weekly change of ventilator tubing for their study participants.

Regarding *Handling of Medication*, three studies described the use of antibiotics [25, 27, 29]. Two studies described the frequency of antibiotics in their study populations [25, 27]. For example, Schwerdtner et al. [27] found that within the last six months before the study was conducted, 75% of SLC residents received antibiotics. Banfi et al. [29] used antibiotics and Albuterol successfully to treat infection related ARF at home.

*Caring for Infected Persons* was mentioned in two studies [28, 31]. Horvath et al. [28] stated that hygiene standards regarding MRSA, MDRO and Norovirus were mostly available in inspected SLC in Munich. Cahill et al. [31] described isolation practices for infection prevention and control in SNF.

With regard to *Staff Hygiene*, two studies described hand hygiene and the availability of personal protective equipment (PPE) in SLC [27, 28]. Aspects regarding *Laundry Hygiene* were addressed by Horvath et al. [28]. For example, their findings showed that inspected SLC had laundry rooms equipped with industrial washing machines. However, there was a need for advice regarding laundry preparation and that working instructions were partly missing.

Recommendations on *Relatives and Visitors*, *Waste Management*, and *Kitchen Hygiene* were not addressed in any of the included studies.

## Discussion

This scoping review of English and German literature mapped the breadth of evidence with regard to hygiene management for ventilated persons in the home care setting. All in all, evidence could not be found for all aspects of hygiene management in outpatient care according to KNAIB [20]. While most evidence was found for the domains *Infectious Critical Activities*, *Quality Management for Hygiene* and *Training and Education*, evidence gaps regarding *Kitchen Hygiene*, *Relatives and Visitors* and *Waste Management* could be identified. Thus, underlying research questions of this study regarding hygiene measures recommended for both relatives and ventilated patients themselves remain unanswered.

Concerning measures of hygiene management recommended for relatives and visitors of ventilated patients, no evidence could be identified. Nevertheless, there is evidence that tensions and ambiguities between relatives and caregivers regarding decision-making can occur, especially in the home care setting [32, 33]. A qualitative study among health care assistants of ventilated patients showed that the work setting "home" is seen as challenging [33]. The handling of complex medical technologies in the home environment requires carefulness about hygiene as well as awareness of infections concerning the HMV [33]. Moreover, family members try to control treatment decision-making and space, as it is their home [32]. This may lead to conflicts especially when nurses apply practices unknown to the relatives [33]. Hence, disputes with relatives about performing tasks could hinder health care assistants performing care [33]. The clarification about the division of tasks and responsibilities could help to avoid tensions [34]. Therefore, the involvement of patients and their relatives in care-related communication is necessary, especially in the home care setting; information sharing and relationship building are considered crucial for safe care [17]. Communication can be supported by, for instance, written educational materials [17].

Communication with relatives is also recommended regarding the handling of medication*.* The present review revealed the relevance of antibiotics in the current literature on HMV in home care [25, 27, 29]. Antibiotics as part of the treatment of respiratory tract infections are used rather frequently in HMV patients [27, 29]. Considering the emergence of MRSA, the correct use of antibiotics is of great relevance in all care settings [35]. Van Huizen et al. [36] found evidence for providing education for nurses regarding the relation of antibiotics and their antimicrobial stewardship. Besides documentation and medication plans, the inclusion of relatives and patients in medication management is considered an important aspect of patient safety [17].

This review found no evidence regarding waste management in outpatient care. In inpatient care settings, Hansen et al. found [15] deficient waste management in nursing homes in Germany. For example, in the majority of homes, there was no risk assessment on waste and written instructions for waste disposal were incomplete [15]. Moreover, there is a lack of both protective personal equipment and handwashing stations for waste removal staff [15]. Ikeda et al. [37] examined the status of home medical waste collection in Japan and found that more than 50% of the home medical care nurses collected hazardous waste such as syringes and needles. In contrast, the collection rate for non-hazardous waste, such as urinary catheters, tracheal suction catheters, nasal masks, was lower [37]. This suggests that such waste is more often disposed of with normal household waste. In a previous study, Ikeda et al. [38] considered the nurses’ education as a key factor for patient education, because nurses teach their patients the proper storage of waste, waste segregation, and disposal. When nurses knew the waste management guidelines, patients’ education status improved [38]. Again, communication about responsibilities is crucial for providing a patient’s safe environment. Thus, only educated personnel should collect infectious critical and hazardous medical waste disposal [38]. This is relevant, especially for MRSA-contaminated material. On spatial requirements, Matos et al. [39] found that places for storing waste by groups or external storage installations are a prerequisite for adequate waste management. Thus, when care is carried out in facilities that are not appropriate for medical care, inadequate practices can lead to occupational accidents caused by infectious material [39]. Considering this, waste management in SLC must be critically evaluated. Schwerdtner et al. [27] found missing structural and technical requirements in SLC in Germany, and therefore, evaluated the buildings as inappropriate for medical care. This confirms the findings reported by Gleich et al. [10] on the appropriateness of conventional rented facilities or apartments for medical care.

Concerning waste management and handling of medication, nurses’ responsibility has already become clear. Due to their intense relationship with patients and relatives, they represent important contact persons, and are, therefore, considered an important source of patient education [32, 33, 38, 40]. Fundamental to this is solid knowledge. Although findings of this review report on training and education for both nurses and patients in different care settings [24, 27, 28, 31], knowledge gaps in some areas of hygiene management were revealed. In the handling of medical devices, Schwerdtner et al. [27] and Horvath et al. [28] found inappropriate cleaning techniques of tracheal cannulas. Furthermore, a need for advice regarding laundry preparation was identified in SLC, which is in line with findings in the setting of inpatient care [15, 28]. Regarding the organisation of educational programmes, research repeatedly points out the importance of on-the-job-trainings and supervision models in HMV [40–42].

Another research gap revealed is missing evidence on kitchen hygiene in the context of HMV. However, it is already known that food-borne infections can be caused by contamination of kitchen surfaces, refrigerators, and hands [43, 44]. In their review on Clostridium difficile in domestic environments, Warriner et al. [45] found that food can be contaminated during preparation and handling. This is potentially hazardous to immunocompromised patients. Even in the hospital environment, the occurrence of Staphylococcus bacteria in kitchen equipment was shown [46]. Taché et al. [43] stated in their review on hygiene in the home kitchen that settings not under the control of competent authority are at higher risk for food-borne infectious diseases.

Regarding implemented practices and programmes for infection prevention and control, this review found hygiene plans, compulsory hygiene management programmes, and qualified nursing personnel for quality management of hygiene [26–28, 31]. Although standards for ventilation-specific activities such as suctioning or cleaning of tracheal cannulas exist, a lack of appropriate skills among nursing personnel was found [28]. Moreover, standards for MRSA, MDRO, and Norovirus were partly missing in SLC [28]. In some cases, employees did not have access to these documents [28]. Regarding the lack of standards in outpatient care, Adler et al. [47] stated that standards for MDRO were missing in all audited nursing services in Bavaria. In particular, the lack of awareness of the issue, a lack of nurses’ knowledge, and the lack of legal regulations are reasons for this [47]. To address this problem, access to hygiene plans needs to be ensured, for example by making them available on the intranet [15]. Moreover, the adaption of hygiene plans to local conditions could help to improve the implementation into clinical practice [15, 35]. Current evidence shows that applying guidelines from evidence in hospitals to home health care is likely to be inappropriate [35]. Because homecare represents a complex care setting, common infection prevention and control processes may not be possible. This is the case, for example, in the diagnosis of infections [35].

Cahill et al. [31] reported ICP working in SNF responsible for quality management on hygiene. Most ICPs were qualified by having at least an associate degree in “nursing education”, but seldom had a bachelor’s degree. It must be considered that the study was conducted in 1997 and that bachelor’s degrees in the field of health care service are more common today. What is still relevant though is the aspect that the majority of questioned ICP are responsible for at least another non-infection control related position [31]. These findings are in line with Shang et al. [35, 48] who repeatedly reported that professionals responsible for infection prevention in home health care mostly have various responsibilities apart from infection prevention, and moreover, are not certified full-time infection control practitioners.

This review revealed a considerable relevance of infectious critical activities in dealing with MRSA and MDRO [25–27, 30, 31]. This is not unexpected, since MRSA colonisation of HMV is estimated to be high [10]. For infection prevention and control, included studies in this review described isolation practices, annual screenings and screenings of new admissions on MDRO [27, 31]. However, Schwerdtner et al. [27] found that screenings and annual swabs are not performed systematically in SLC. This could be evaluated critically in terms of proven high MRSA-colonisation rates in outpatient care [10].

### Strengths and limitations

To the authors’ knowledge, this is the first scoping review of international literature on hygiene management in outpatient intensive care. Three relevant databases, PubMed, CINAHL, and Web of Science were searched using a systematic search strategy. Thus, this study provides a comprehensive overview of the current state of research, summarizing existing evidence and identifying research gaps regarding hygiene management in HMV. The transparent methodology in accordance with the PRISMA guideline for scoping reviews ensures the reproducibility of the included literature and the presentation of results. However, some limitations also need to be discussed. A remarkable aspect is that despite deliberately keeping the inclusion and exclusion criteria broad, only quantitative studies could be included for analysis. Moreover, not limiting the publication years, it must be considered that the management of MDRO and MRSA has become more relevant in the last 20 years. A critical appraisal and evaluation of the methodological quality was not performed, although this is not necessary for scoping reviews [21]. However, as a wide range of study types were included, there was a possibility to include studies with weak methodology. In addition, some of the studies included have small sample sizes and investigated specific diseases. This should be taken into account when interpreting the evidence found. As this review revealed a wide range of prevalence data for HMV, future research could focus on elaborating existing data sets concerning the prevalence rates in a meta-analysis, for example.

## Conclusion

This scoping review on hygiene management for long-term ventilated persons in outpatient care revealed research gaps regarding evidence in kitchen hygiene, relatives and visitors, and waste management. Based on current literature, research questions underlying this review could not be entirely answered. Infection control programmes included qualified personnel, hygiene plans, and standards for MRSA and MDRO. However, the literature yielded a lack of comprehensive implementation of hygiene measures into practice. As an implication for practice, it can be concluded that the appropriateness of hygiene plans for outpatient care settings must be ensured. Moreover, training and education modalities such as on-the-job training and supervision are crucial. Binding legal requirements and audits may help regulate the implementation of hygiene measures.

## Supplementary Information

**Additional file 1.**

**Additional file 2.**

**Additional file 3.**

## Data Availability

All data generated or analysed during this study are included in this published article and its supplementary information files.

## Abbreviations

ARF: Acute respiratory failure
AWMF: Association of Scientific Medical Societies (*Arbeitsgemeinschaft Wissenschaftlich-Medizinischer Fachgesellschaften*)
CDI: Clostridium difficile
CRE: Carbapenem-resistant Enterobacteriaceae
EVA: Expiratory valve
FSC: Flat-sharing community
GP: General practitioner
HMV: Home mechanical ventilation
HVC: Home ventilation circuit
ICP: Infection control practitioner
IPReG: Law to Strengthen Intensive Care and Medical Rehabilitation *(Intensivpflege- und Rehabilitationsstärkungsgesetz)*
MDK: Medical Service of Health Insurance *(Medizinischer Dienst der Krankenkassen)*
MDRO: Multidrug-resistant organisms
MRSA: Methicillin-resistant Staphylococcus aureus
PPE: Personal protective equipment
PRISMA: Preferred Reporting Items for Systematic Reviews
RGU: Department of Health and Environment *(Referat für Gesundheit und Umwelt)*, Munich
RKI: Robert-Koch-Institute
SLC: Shared living community
SNF: Skilled nursing facility
VAP: Ventilator associated pneumonia
VRE: Vancomycin-resistant enterococci
