# Correction to: Hygiene management for long-term ventilated persons in the home health care setting: a scoping review

**DOI:** 10.1186/s12913-022-07824-7

**Published:** 2022-03-28

**Authors:** Isabel Hoeppchen, Carola Walter, Stefanie Berger, Anna Brandauer, Nicole Freywald, Patrick Kutschar, Katharina Maria Lex, Annemarie Strobl, Irmela Gnass

**Affiliations:** Institute of Nursing Science and Practice, Paracelsus Medical University Salzburg, Strubergasse 21, Salzburg, Austria

**Correction to: BMC Health Serv Res 22, 244 (2022)**

**https://doi.org/10.1186/s12913-022-07643-w**

Following publication of the original article [1], an error was identified in reference 27.

Originally this reference was included as: Schwerdtner N, Dietsch S, Trommer S, Weise A. Infektionsprävention/ Herausforderung MRE in außerklinischen Intensivpfege-WGs [Infection prevention/ challenge of MDRO in outpatient intensive care fat-sharing communities]. Das Gesundheitswesen. 2019;81(03):P18.

Reference 27 needs to be corrected to: Schwerdtner N-L, Trommer S, Dietsch S, Stein C, Weise A, Popp A, Kipp F. Herausforderungen im Umgang mit MRE in außerklinischen Intensivpflege-Wohngemeinschaften. Erfahrungsbericht und Ergebnisse einer Prävalenzerhebung zu multiresistenten Erregern im Stadtgebiet Jena. Epid Bull. 2020;37:3–11. 10.25646/7042.

The original article [1] has been corrected.

## References

[1] Hoeppchen (2022). Hygiene management for long-term ventilated persons in the home health care setting: a scoping review. BMC Health Serv Res.

