# Supplementary material for: Hygiene management for long-term ventilated persons in the home health care setting: a scoping review
Source: BMC Health Serv Res. 2022 Feb 23;22:244. doi: 10.1186/s12913-022-07643-w (PMC8864850; doi:10.1186/s12913-022-07643-w)
Supplement: Supplementary file 1 — Additional file 1. [file 12913_2022_7643_MOESM1_ESM.pdf]

Appendix 1: Full search strategy for CINAHL. Hygiene Management for Long-term Ventilated Persons in the Home Health Care Setting: A Scoping Review

Search date: 22.07.2020

Search number	Mesh term, text word and combinations	Results
	**Home mechanical ventilation**	
**1**	Artificial respiration*	12,341
**2**	Respiration artificial	21,610
**3**	Mechanical ventilation*	35,977
**4**	Home mechanical ventilation	417
**5**	HMV	199
**6**	Invasive ventilation	2,949
**7**	Noninvasive ventilation	3,217
**8**	Long term ventilation	459
**9**	Home care ventilation	16
**OR**	1-9	**44,816**
	**Hygiene management**	
**10**	Hygiene	105,077
**11**	Hygiene management	194
**12**	Hand hygiene	11,694
**13**	Infection*	552,791
**14**	Infection prevention	73,483
**15**	Infection control	68,730
**16**	Health care associated infection	25,013
**OR**	10-16	621,634
**17**	MRSA	14,939
**18**	Methicillin-Resistant Staphylococcus aureus	12,613
**AND**	17-18	8,842
**OR**	10-16 OR 17-18	**622,287**
	**Setting**	
**19**	Home	605,744
**20**	Home health nursing	610
**21**	Home nursing	26,808
**22**	Home care service*	20,267
**23**	Home care agencies	5,290
**24**	Home care aides	1,120
**25**	Communal living arrangements	66
**26**	Small scale living	41
**27**	Flat	38,504
**38**	Outpatient	198,309
**29**	Homebo#nd person*	55
**OR**	19-29	**769,475**
	**exclusion under 18 years incl. ICU**	
**30**	Neonate*	42,085
**31**	children	1,491,337
**32**	Adolescents	666,953
**33**	Pediatric*	566,434
**OR**	30-33	1,635,014
**34**	ICU	155,522
**35**	Intensive care unit	132,597
**AND**	34-35	133,249
**OR**	30-33 OR 34-35	**1,703,425**
**AND NOT**	Home mechanical ventilation AND Hygiene management AND Setting NOT exclusion under 18 years incl. ICU	**661**

Appendix 1: Full search strategy for CINAHL. Hygiene Management for Long-term Ventilated Persons in the Home Health Care Setting: A Scoping Review

Search term
(Artificial respiration* OR Respiration artificial OR Mechanical ventilation* OR Home mechanical ventilation OR HMV OR Invasive ventilation OR Noninvasive ventilation OR Long term ventilation OR Home care ventilation) AND (Hygiene OR Hygiene management OR Hand hygiene OR Infection* OR Infection prevention OR Infection control OR Health care associated infection OR (MRSA AND Methicillin-Resistant Staphylococcus aureus)) AND (Home OR Home health nursing OR Home nursing OR Home care service* OR Home care agencies OR Home care aides OR Communal living arrangements OR Small scale living OR Flat OR Outpatient OR Homebo#nd person*) NOT (neonate* OR children OR adolescent OR pediatric* OR (ICU AND intensive care unit))
