# Supplementary material for: Hygiene management for long-term ventilated persons in the home health care setting: a scoping review
Source: BMC Health Serv Res. 2022 Feb 23;22:244. doi: 10.1186/s12913-022-07643-w (PMC8864850; doi:10.1186/s12913-022-07643-w)
Supplement: Supplementary file 2 — Additional file 2. [file 12913_2022_7643_MOESM2_ESM.docx]

| Data charting sheet - for health care professionals, patients, relatives |
| --- |
| Study description |
| First author |
| Title |
| Year |
| Country |
| Study aims |
| Methodology/ Measures |
| Setting and Participants |
| Sample size |
| Sex |
| Age of participation |
| Kind of disease |
| Kind of artificial ventilation (incl. duration) |
| Family participation |
| Concept of home-based setting/ Licensed beds |
| Professions/ Qualifications involved/ Duration of professional employment |
| Cooperation (e. g. lung specialist, Weaning centre) |
| Key Findings - Hygiene management |
| Quality management for hygiene, e. g. infection control practitioner, h for infection prevention and control |
| … - Training and Education (incl. in-/ outdoor training) |
| … - Staff Hygiene (incl. PPE, Hand Hygiene) |
| … - Relatives/ Visitors (incl. physicians/ therapists) |
| … - Cleaning and disinfection aspects |
| … - Handling of medical devices |
| … - Waste management |
| ... - Infectious critical activities (incl. MRSA, Screening, Surveillance) |
| … - Caring for infected persons |
| … - Handling of medication |
| … - Laundry hygiene |
| … - Kitchen hygiene |
| Conclusion/ Limitations |
