# Supplementary material for: Hygiene management for long-term ventilated persons in the home health care setting: a scoping review
Source: BMC Health Serv Res. 2022 Feb 23;22:244. doi: 10.1186/s12913-022-07643-w (PMC8864850; doi:10.1186/s12913-022-07643-w)
Supplement: Supplementary file 3 — Additional file 3. [file 12913_2022_7643_MOESM3_ESM.pdf]

Appendix 3: Data Charting Sheets. Hygiene Management for Long-term Ventilated Persons in the Home Health Care Setting: A Scoping Review

All articles in alphabetical order

Data charting sheet - for health care professional, patients, relatives	
**Study description**	
**First author**	Banfi, Paolo
**Title**	Home Treatment of Infection-Related Acute Respiratory Failure in Kyphoscoliotic Patients on Long-Term Mechanical Ventilation
**Year**	2007
**Country**	France
**Study aims**	To study the possibility of home treatment of infection-related ARF in kyphoscoliotic patients on long term mechanical ventilation
**Methodology/Measures**	**Study design:** prospective cohort study<br><br>**Study period:** 1998-2002<br><br>**Study inclusion:** Tachypnea >30 breaths/min, Cyanosis, Purulent secretions, Cough, Fever<br><br>**Inclusion process:**<br>- Chest specialist, GP + nurse examined patient at home: Arterial blood samples, ECG readings + expectorated sputum samples<br>- MV was administered continuously until the morning + pulse oximetry was recorded all night → if patient showed improvement in arterial blood gas values 1 hour after initiating mechanical ventilation + mean nocturnal pulse oximetry was >90%, patient was invited to the home treatment protocol<br><br>**Treatment of included patients:**<br>- progressively decrease daily duration of MV according to their tolerance of spontaneous breathing under supervision of med. staff → Treatment with antibiotics + Albuterol inhaler *(s. cat. "Handling of medication")*<br><br>**Hypothesis:**<br>- Home care of ARF can be as safe + effective as hospital care<br><br>**Measures:**<br>- 1x/ week: arterial blood samples were collected after 1 hour of spontaneous breathing without MV

	- 2 weeks after starting antibiotics: sputum collection + culture
**Setting, Participants**	
**Sample size**	**7 patients**<br><br>(Background: between 1998-2002 **8/195 patients** within the program of long-term MV had an infection-related ARF + were therefore eligible for this study; 1 was excluded because he refused treatment at home)
**Sex**	3 women, 5 men
**Age of participation**	61 years (mean)
**Kind of disease**	- Idiopathic severe kyphoscoliosis (Cobb angle >70°)<br>- Developed infection-related ARF during a 4 year period
**Kind of artificial ventilation (incl. duration)**	**All patients** had mechanical ventilation for 31 months (mean)<br>- **7/8** NPPV + commercial mask<br>- **3/8** volume-assist ventilator<br>- **4/8** pressure assist ventilator + circuit that includes an expiratory valve (backup rate of mean 16 breaths/min)<br>- **1/8** invasively ventilated<br><br>**During study**:<br>- Nearly continuous MV (>20h/d) for the first week with ventilator settings used in their baseline condition<br>- Progressively decreasing daily duration of mechanical ventilation according to tolerance of spontaneous breathing
**Family participation**	Not specified
**Concept of home-based setting/Licensed beds**	At the patient's home
**Professions/Qualifications involved/Duration professional employment**	Staff involved in the study: **Nurse**, **GP** + **chest specialist** evaluated + supervised the patient at home<br>- Nurse: visited the patient's home 3x/d<br>- GP: visited patient 2x/week<br>- No emergency visit was necessary
**Cooperation (e.g. Lung specialist, Weaning centre)**	Not specified
**Key Findings - Hygiene management**	
**Quality management for hygiene, e.g. Infection control practitioner, duration h for infection prevention and control**	
**… - Training /education (incl. in- outdoor training)**	Not specified
**… - Staff hygiene (incl. PPE, Hand hygiene)**	Not specified
**… - Relative/visitors (incl. Physicians/Therapists)**	Not specified

**… - Cleaning and disinfection aspects**	Not specified
**… - Handling of medical devices**	
**… - Waste management**	Not specified
**... - Infectious critical activities (incl. MRSA, Screening, Surveillance)**	Not specified
**… - Caring for persons (prevention and infection control)**	Not specified
**… - Handling of medication**	**Antibiotics**<br>- All patients<br>- Duration: 2 weeks<br>- Dose: Clarithromycin 500mg 2x/d; intramuscular ceftazidime 1g 3x/d<br><br>**Albuterol**<br>- Metered-dose-inhaler during MV<br>- 4x/d
**… - Laundry hygiene**	Not specified
**… - Kitchen hygiene**	Not specified
**Conclusion/Limitations**	

Conclusions:
- 7/7 kyphoscoliotic patients on long-term MV suffered from infection-related ARF + were successfully treated at home
- Treatment with antibiotics + NPPV (in 6/7 cases)
- Increase of daily duration of MV without any change of ventilator settings was well tolerated by patients (familiarity)
- Cost-saving treatment at home: average duration of hospitalization of these patients is 2-3 weeks

Limitation: no control group

Notes for review:
- No hygiene-management
- Specific group of patients
- Main outcome: infection-treatment at home is feasible, efficient + cost-effective

Abbreviations:

ARF = acute respiratory failure
GP = general practitioner
ECG = electrocardiogram
NPPV = noninvasive positive pressure ventilation
MV = mechanical ventilation

Data charting sheet - for health care professional, patients, relatives	
**Study description**	
**First author**	Cahill, Christine K.
**Title**	California Skilled Nursing Facilities – A Survey of Infection Prevention and Control Practices and Educational Needs
**Year**	1997
**Country**	USA
**Study aims**	- To assess characteristics of SNF infection prevention + control programs<br>- To determine policies for admitting + placing of MRSA-/ VRE-colonized patients<br>- To determine educational needs of the respondents possible for infection prevention + control programs in SNF
**Methodology/Measures**	**Study design:** observational/ descriptive study<br><br>**Method:** quantitative survey<br>- Among ICPs in all Californian SNFs<br>- Survey included 3 parts: facility biographical profile, antibiotic resistant microorganism profile + educational needs assessment<br>→ a total of 1.454 surveys were mailed to free-standing + hospital-based SNF (no reminders)
**Setting, Participants**	
**Sample size**	444 SNF (response rate 30.5%)<br>- 283 (63.7%) free-standing<br>- 161 (23.2%) hospital-based<br>- 58 (13.1%) other (veterans administration or not specified)
**Sex**	Not specified
**Age of participation**	Not specified
**Kind of disease**	Not specified
**Kind of artificial ventilation (incl. duration)**	Not specified
**Family participation**	Not specified
**Concept of home-based setting/Licensed beds**	Skilled nursing facilities (SNF)<br>- Hospital-based distinct-part (HB) or free-standing (FS)<br>- Licenced beds: 10 to >350; most SNF (68%) < 99 beds<br>*(more details s. Tab 1+2)*
**Professions/Qualifications involved/Duration professional employment**	(see table below)

	Hospital-based SNF	Free-standing SNF
ICPs	- 88/102 (86.3%) associate degree or higher in “nursing education”	- 203/278 (73%) associate degree or higher in “nursing education”

<table>
<tr><td></td><td>
<table>
<tr><td></td><td>- 39/88 (38.2%) bachelor’s degree in “nursing”
- ??/?? (13.7%) licensed “vocational nurses”</td><td>- 79/278 (28.4%) bachelor’s degree in “nursing”
- 75/278 (27%) licensed “vocational nurses”</td></tr>
<tr><td>Responsibilities</td><td>-79/102 responsible for at least one other non-infection –control-related position (Director Staff Development 27.8%; Employee Health Nurse 16.5%)</td><td>- 236/278 responsible for at least one other non-infection –control-related position (Director Staff Development 43.6%; Director Nursing Administration 26.7%)</td></tr>
<tr><td>ICP-experience (s. Tab. 4)</td><td>- 54/96 (56.2%) ICP < 5 years
- 19/96 (19.8%) ICP < 1 year</td><td>- 184/265 ICP < 5 years
- 72/26 ICP < 1 year</td></tr>
<tr><td>Memberships</td><td>- 59/99 membership APIC
- 27/59 certified by CBIC</td><td>- 41/269 (15%) membership APIC
- 7/41 (17%) certified by CBIC</td></tr>
</table>
</td></tr>
<tr><td>Cooperation (e.g. Lung specialist, Weaning centre)</td><td>Not specified</td></tr>
<tr><th>Key Findings - Hygiene management</th><th></th></tr>
<tr><td>Quality management for hygiene, e.g. Infection control practitioner, duration h for infection prevention and control</td><td>
<table>
<tr><th></th><th>Hospital-based SNF</th><th>Free-standing SNF</th></tr>
<tr><td>Average h/week for infection control + prevention activities</td><td>- 35/97 (36.1%) < 5h
- 22/97 (22.7%) < 2h</td><td>- 127/267 (47.7%) < 5h
- 58/267 (21.7%) < 2h</td></tr>
<tr><td>ICPs</td><td colspan="2">Available</td></tr>
<tr><td>Programs</td><td colspan="2">Infection Prevention + Control Program (required by Health Care Financing Administration; HCFA)
- Staff hygiene
- Isolation procedures</td></tr>
</table>
</td></tr>
<tr><td>… - Training /education (incl. in- outdoor training)</td><td>Educational needs for infection prevention + control rated by ICPs:
- New employee + annual education programs: Hand washing (87.3%), pathogens standard (84.6%) + tuberculosis control program (83.6% of ICPs rated 4 = very important)
- Employee health: tuberculosis screening (76.7%)</td></tr>
</table>

<table>
<tr><td></td><td>- Resident's Health: tuberculosis screening for new admission (76.9%)
- Antibiotic-Resistant Microorganisms: admitting patients with MRSA/ VRE (81.4%)
- Antibiotic Utilization Program: appropriate use of antibiotics (80.6%)
- Microbiology: colonisation vs. infection (75.7%)
- Surveillance Program: collecting, analysing + reporting data (74.6%)
- Isolation Precautions: standard isolation precautions (80.1%)
(topics from APIC-Guideline for infection prevention + control) (for more subtopics s. Tab. 5)</td></tr>
<tr><td>… - Staff hygiene (incl. PPE, Hand hygiene)</td><td>Not specified</td></tr>
<tr><td>… - Relative/visitors (incl. Physicians/Therapists)</td><td>Not specified</td></tr>
<tr><td>… - Cleaning and disinfection aspects</td><td>Not specified</td></tr>
<tr><td>… - Handling of medical devices</td><td>Not specified</td></tr>
<tr><td>… - Waste management</td><td>Not specified</td></tr>
<tr><td>… - Infectious critical activities (incl. MRSA, Screening, Surveillance)</td><td>
<table>
<tr><td></td><td>Hospital-based SNF</td><td>Free-standing SNF</td></tr>
<tr><td>Isolation practice</td><td>- 14/96 (14.6%) no private room (PR)
- 38/96 (39.6%) < 3 PR
- 44/96 (45.8%) > 4 PR
- 90.9% use PR for isolation</td><td>- 73/260 (28.1%) no private room (PR)
- 107/260 (41.1%) < 3 PR
- 80/260 (30.8%) > 4 PR
- 74.5% use PR for isolation</td></tr>
<tr><td>Admissions</td><td>- do not culture new admission for MRSA (87.5%) or VRE (90.6%)</td><td>- do not culture new admission for MRSA (90.5%) or VRE (93.4%)</td></tr>
</table>
</td></tr>
<tr><td>… - Caring for persons (prevention and infection control)</td><td>
<table>
<tr><td></td><td>Hospital-based SNF</td><td>Free-standing SNF</td></tr>
<tr><td>Dealing with MRSA (VRE) colonised</td><td>- 58% (73.8%) would place in PR
- 71.2% (44.4%) would place with roommate without invasive devices</td><td>- 31.9% (47.6%) would place in PR
- 87.5% (72.8%) would place with a roommate without invasive devices</td></tr>
<tr><td>Dealing with MRSA (VRE) infected</td><td>- 82.4% (94%) would place in PR
- 60.3% (39.4%) would place with roommate without invasive devices</td><td>- ?? (71%) would place in PR
- ?? (68.8%) would place with roommate without invasive devices</td></tr>
</table>
</td></tr>
<tr><td>… - Handling of medication</td><td>Not specified</td></tr>
<tr><td>… - Laundry hygiene</td><td>Not specified</td></tr>
<tr><td>… - Kitchen hygiene</td><td>Not specified</td></tr>
<tr><td>Conclusion/Limitations</td><td></td></tr>
<tr><td colspan="2">Conclusions:
- SNFs are required by HCFA to have infection prevention + control programs
- Differences between hospital-based SNF + free-standing SNF with regard to their programs for infection prevention + control
- Very few SNFs reported performing surveillance cultures on all new admissions to detect antibiotic-resistant organisms

Notes for Review:</td></tr>
</table>

- No limitations discussed!
- Article was published in 1997! → topic still relevant but importance of dealing with MDRO has changed
- Setting described is not ambulatory intensive care!
- No focus on ventilated patients

Abbreviations:

APIC = Infection Control and Epidemiology, Inc.

CBIC = Certification Board for Infection Control

HCFA = Health Care Financing Administration

ICP = infection control practitioner

PPE = personal protective equipment

SNF = skilled nursing facilities

VRE = vancomycin-resistant enterococcus

Appendix 3: Data Charting Sheets. Hygiene Management for Long-term Ventilated Persons in the Home Health Care Setting: A Scoping Review

<table>
<tr><th colspan="2">Data charting sheet - for health care professional, patients, relatives</th></tr>
<tr><th>Study description</th><th></th></tr>
<tr><td>First author</td><td>Chenoweth, Carol</td></tr>
<tr><td>Title</td><td>Ventilator-Associated Pneumonia in the Home Care Setting</td></tr>
<tr><td>Year</td><td>2007</td></tr>
<tr><td>Country</td><td>USA</td></tr>
<tr><td>Study aims</td><td>Characterise VAP in HMV-patients
- Rate + incidence of VAP
- Demographic characteristics
- Potential risk factors for VAP
- Outcomes of VAP in this setting</td></tr>
<tr><td>Methodology/Measures</td><td>Study design: Retrospective Cohort Study

Inclusion criteria:
- All patients who received HMV through MedEquip + were followed up by MedEquip respiratory therapists
- Included patients represent 80% of all patients receiving HMV cared by MedEquip
- “Cases” = study patients who developed VAP (cases were ascertained through review of home nursing + respiratory therapy records)

Measures:
Demographic + clinical variables were extracted from MedEquip + UHMS medical records (standardised sheet) by visiting home nurses + respiratory therapists
- Respiratory status
- Sputum
- Oxygen requirements
- Suctioning requirements
- Infections
- Outpatient antibiotic use
→ variables were collected up to the time of onset of VAP

Analysis: statistical analysis (Chi²-/ Fisher exact-test, student t-test, p-value)</td></tr>
<tr><th>Setting, Participants</th><th></th></tr>
<tr><td>Sample size</td><td><table><tr><th>total</th><th>With VAP</th><th>Without VAP</th></tr><tr><td>57 patients</td><td>27</td><td>30</td></tr></table></td></tr>
</table>

Sex

	With VAP	Without VAP
Male	13 (48%)	13 (43%)

Age of participation

	With VAP	Without VAP
Age	19.3 (mean)	14.3 (mean)

Kind of disease

VAP = ventilator-associated pneumonia

- Predominant cause of VAP in hospitalized patients: Staph. aureus + Pseudomonas aeruginosa
- Hospitalisation: 4-11 days (each episode costs >10,000 USD)
- Mortality in ICU: up to 40%
- Multiple episodes of VAP separated >1 month/ with different x-ray findings/ new organisms were considered as independently

Study-results

VAP-rates	- 79 independent episodes in 27/57 (47%) patients<br>- 1.3 VAP-episodes/ patient<br>- 48 (61%) of episodes occurred within the first 500 days of ventilation<br>- 1.55 VAP-infections/ 1000 ventilator-days
Hospitalisation	- 69 (87%) of VAP<br>- Mean duration: 7 +/- 9 days
Culture	- 17 (35%) Pseudomonas<br>- 11 (22%) Staphylococcus aureus<br>(more detail: Tab. 1)

Kind of artificial ventilation (incl. duration)

	With VAP	Without VAP
Ventilator types	Bilevel ventilation to tracheotomy tube, standard pressure control ventilation to tracheostomy tube, external negative-pressure, standard pressure support *(more details: Tab. 2)*	
Ventilation duration	1,012 +/-673 days	781+/- 572 days
	**20/27 (74%)** 17-24h/d	**14/30 (7%)** 17-24h/d
	**All** 57 patients underwent a total of 50.762 days of ventilation (mean duration of ventilation: 890.6 ventilator-days/ patient)	

	**Indication for ventilation:**<br>- Congenital respiratory disease<br>- Obstructive respiratory disorders<br>- Nervous system disease<br>- Myopathies
**Family participation**	Provide patients' primary care (if not: home nursing facilities)
**Concept of home-based setting/Licensed beds**	MedEquip Home Care Services department at the UMHS<br><br>**Background:**<br>- Respiratory services: provision of oxygen for home use, aerosol therapies, tracheostomy supplies, HMV services<br>- Provided services for 12000 respiratory patients since company was found in 1995
**Professions/Qualifications involved/Duration professional employment**	Staff involved in the study:<br>- 4 respiratory therapists (follow up)<br>- 1 nurse<br>- 15 technicans<br><br>Primary care for patients:<br>- Nursing services (if not: family members)
**Cooperation (e.g. Lung specialist, Weaning centre)**	Not specified
**Key Findings - Hygiene management**	
**Quality management for hygiene, e.g. Infection control practitioner, duration h for infection prevention and control**	Not specified
**… - Training /education (incl. in- outdoor training)**	Not specified
**… - Staff hygiene (incl. PPE, Hand hygiene)**	Not specified
**… - Relative/visitors (incl. Physicians/Therapists)**	Not specified
**… - Cleaning and disinfection aspects**	Not specified
**… - Handling of medical devices**	Ventilator tubing was changed weekly for all patients
**… - Waste management**	Not specified
**... - Infectious critical activities (incl. MRSA, Screening, Surveillance)**	Tracheostomy suctioning (clean technique)
**… - Caring for persons (prevention and infection control)**	Not specified
**… - Handling of medication**	Not specified
**… - Laundry hygiene**	Not specified
**… - Kitchen hygiene**	Not specified

Appendix 3: Data Charting Sheets. Hygiene Management for Long-term Ventilated Persons in the Home Health Care Setting: A Scoping Review

Conclusion/Limitations	
**Conclusions:**<br>- VAP-rates + mortality rates are much lower in HMV vs. ICU population<br>- VAP was likely to occur during the first 100 days of HMV (caregiver learning curve for providing ventilator care?)<br>→ reducing risk of VAP should be focused on patients who require ventilation for longer daily durations/ who are new to receiving HMV<br>- No association of VAP with age, sex, underlying disease, reason for/ type of ventilation, antacid therapy, steroid use<br><br>**Limitations:**<br>- Cohort from a homogeneous population from a university-affiliated single home care service<br>- Very young patients (less comorbidities than patients in ICU)<br>- Only 33/ 79 (42%) VAP-episodes had culture results available<br><br>**Notes for review:**<br>- no hygiene management, but infection control + home treatment of VAP<br>- young patients (doesn’t meet our inclusion criteria)	

Abbreviations:

HMV = home mechanical ventilation

ICU = intensive care unit

UMHS = University of Michigan Health System

VAP = ventilator associated pneumonia

Appendix 3: Data Charting Sheets. Hygiene Management for Long-term Ventilated Persons in the Home Health Care Setting: A Scoping Review

Data charting sheet - for health care professional, patients, relatives	
**Study description**	
**First author**	Horvath, Leila
**Title**	Surveillance of Supervised Flat-Sharing Communities Requiring Intensive Home Care: Results and Conclusions
**Year**	2018
**Country**	Germany
**Study aims**	- Overview of the type of care provided in outpatient IC-FSC in Munich<br>- Review implementation of hygiene + emergency management<br>- Advice + support if necessary
**Methodology/Measures**	**Study Design:** Observational/ descriptive study<br><br>**Method:**<br>a. **Structure** Analysis (Audit in nursing services' offices)<br><br>b. **Inspection** of outpatient FSC (standardised checklists prepared by RGU)<br><br>c. Final **discussion** with care services + review report (defects, complaints + recommendations)<br><br>**Contents of inspection**: Structural data, organisation, client profiles, hygiene management, handling of medical devices/ medication/ narcotics, medical care, emergency management, client safety<br><br>**Review period**: April 2015 - August 2016<br><br>**Data Analysis**: descriptive (excel)
**Setting, Participants**	
**Sample size**	**18/20** outpatient IC- FSC in Munich (*)<br>- A total of 136 care places, of which 85 (62.5%) were occupied at the time of the review<br><br>(*) exclusion criteria for 2 facilities: Renovation or new opening
**Sex**	Not specified
**Age of participation**	Not specified
**Kind of disease**	Not specified
**Kind of artificial ventilation (incl. duration)**	Not specified
**Family participation**	Not specified
**Concept of home-based setting/Licensed beds**	**In general:**

	- Self-determined FSC<br>- Max. 12 residents<br>- Nursing services are freely selectable + have guest status<br><br>**Present study:**<br>- 9 nursing services took care of 20 FSC for outpatient IC in Munich
**Professions/Qualifications involved/Duration professional employment**	- **7/9** Nursing specialist with further training for intensive care in a leading position<br>- **1/9** Nursing service management with home respiration course
**Cooperation (e.g. Lung specialist, Weaning centre)**	- **2/9 care provider**: connected to Weaning-Centre<br>- **3/9** cooperation with external hygiene specialist
**Key Findings - Hygiene management**	
**Quality management for hygiene, eg Infection control practitioner, duration h for infection prevention and control**	- **8/9 care provider**: hygiene representative<br><br>- **18/18 FSC**: hygiene plans on site<br>- **14/18** hygiene plans available for employees<br>- **10/10** hygiene plans complete<br>- **17/18** standard for endotracheal suctioning + handling of tracheal cannulas
**… - Training /education (incl. in- outdoor training)**	- **9/9 care providers**: proven annual hygiene training<br>- **8/9** training documents in hygiene
**… - Staff hygiene (incl. PPE, Hand hygiene)**	Hand hygiene:<br>- **18/18 FSC**: VAH-listed hand disinfectants + wall-mounted hand disinfectant dispensers in client rooms<br>- **14/18** correct hand hygiene (**4/18** complaints: jewellery, watches, painted nails)<br>- **15/18** Hand disinfectant dispensers in rooms in which reprocessing of tracheal cannulas + suction is performed<br><br>PPE:<br>- **18/18** wearing PPE<br>- **18/18** keeping PPE
**… - Relative/visitors (incl. Physicians/Therapists)**	Not specified
**… - Cleaning and disinfection aspects**	- **18/**18 FSC: VAH-listed surface disinfectants (**8/18** correct dosage)<br>- **7/18** VAH-listed fast disinfectant<br>- **1/18** exclusively alcoholic products for surface disinfection<br>- **8/18** tissue dispenser systems (handling correct in **2/8**)
**… - Handling of medical devices**	- **4/18 FSC**: proper preparation of the tracheal cannulae<br>- **18/18** tube + filter change interval according to manufacturer's instructions

	- **14/18** daily functional test of all active medical devices
**… - Waste management**	Not specified
**... - Infectious critical activities (incl. MRSA, Screening, Surveillance)**	Not specified
**… - Caring for persons (prevention and infection control)**	- **14/18 FSC** standard to MRSA<br>- **11/18** standard to MRGN<br>- **13/18** standard to Norovirus
**… - Handling of medication**	Not specified
**… - Laundry hygiene**	- **18/18 FSC**: own laundry<br>- **13/18** industrial washing machines<br>- **18/18** disinfectant detergent<br>- **9/18** work instructions for laundry preparation<br>- **16/18** disinfecting treatment + storage of work clothes<br>- **9/18** improper preparation, drying, storage of cleaning utensils
**… - Kitchen hygiene**	Not specified
**Conclusion/Limitations**	

Conclusions in general: regular consultations, continuing education + hygiene inspections by public health department have positive effects on hygiene + emergency management

Personal qualification: Professional background + continuing education of nurses very different

Hygiene Management

- All care providers had hygiene representatives, but certificates of qualification with different information on scope + duration of the courses
- None of the hygiene representatives had a qualification according to the DGKH guidelines
- Hygiene plans: standards on MRE, MRSA, MDRO were partially missing
- Hand hygiene: well implemented
- Laundry preparation: need for advice; use of small industrial washing machines recommended
- Complaints in handling PPE, reprocessing of tracheal cannulae, respiratory/aspiration accessories + when handling surface disinfectants

→ good test result regarding organisational structures + hygienic equipment
→ So far, there are no binding regulations on the form of care in FSC, even if they are an integral part of outpatient medical care

Notes for review:

- Setting **FSC** applies to our research topic

- Limited data (only Munich)<br>- No limitations discussed by authors<br>- Consider new IPEG

Abbreviations:

DGKH = Deutsche Gesellschaft für Krankenhaushygiene

IC = intensive care

RGU = Referat für Gesundheit + Umwelt

VAH = Verbund für Angewandte Hygiene e.V.

<table>
<tr><th colspan="2">Data charting sheet - for health care professional, patients, relatives</th></tr>
<tr><th>Study description</th><th></th></tr>
<tr><td>First author</td><td>Leonhard, Matthias</td></tr>
<tr><td>Title</td><td>Microbiological evaluation of different reprocessing methods fur cuffed and un-cuffed tracheostomy tubes in home-care and hospital setting</td></tr>
<tr><td>Year</td><td>2016</td></tr>
<tr><td>Country</td><td>Austria/ Great Britain (remains unclear)</td></tr>
<tr><td>Study aims</td><td>- To evaluate different reprocessing methods
- To determine mechanical integrity + functionality of tracheostomy tubes following reprocessing</td></tr>
<tr><td>Methodology/Measures</td><td>Study design: Experimental study (randomised)

Method:
Examination of un-cuffed + cuffed tubes:
- Un-cuffed tubes were changed + collected after minimum of 1 day of use
- Cuffed tubes were changed + collected after minimum of 3 days of use

1. Microbiological assessment
- Microbial concentration on the inner surface of the tubes was assessed BEFORE + AFTER manual cleaning:
• Procedure method A + D: Tube’s lumen was rinsed with 10mL of sterile 0.9% saline solution
• Procedure method B + C: Tube’s lumen was rinsed with validated neutralizers
- Microbial concentration of rinsing solution was determined by using the standard microbiological serial dilution method + planting on different cultures

2. Tubes were assigned to 3 additional (!) cleaning procedures randomly

<table>
<tr><th colspan="3">Cleaning Methods</th></tr>
<tr><td>A</td><td>Manual cleaning</td><td>- Brushing with a new tube brush (available from tube manufacturers)
- Rinsing with tap-water
- Until macroscopically clean result was achieved</td></tr>
</table>
</td></tr>
</table>

B	Manual pre-cleaning + chemical disinfection	- Complete submersion of the cleansed tubes in a commercially available + by manufacturer recommended disinfection solution<br>- For 1h at room temperature<br>- Rinsing with tap-water
C	Manual pre-cleaning + machine-based thermal disinfection in dishwasher	- Tubes were placed vertically in dishwasher<br>- Peak process temp. 65°, standard washing program (48min), no additional cleaning agents
D	Manual pre-cleaning + ultrasound sonification	- US-cleaning in a commercially available US-household device<br>- Rinsing with tap-water

3. Evaluating material integrity

- Cleaned + reprocessed tracheostomy tubes were examined for visible signs of material alteration including colour changes, deformation or other causes for loss of tube-function
- Cuff integrity was tested by inflation at 25mmHg of air + documentation of the maintenance of pressure during 20min with a cuff pressure manometer

**Setting, Participants**	
**Sample size**	16 polymer tracheostomy tubes (8 cuffed + 8 un-cuffed) from hospital in-patients
**Sex**	Not specified
**Age of participation**	Not specified
**Kind of disease**	Not specified
**Kind of artificial ventilation (incl. duration)**	Not specified
**Family participation**	Not specified
**Concept of home-based setting/Licensed beds**	Not specified
**Professions/Qualifications involved/Duration professional employment**	Not specified
**Cooperation (e. g. Lung specialist, Weaning centre)**	Not specified
**Key Findings - Hygiene management**	
**Quality management for hygiene, e. g. Infection control practitioner, duration h for infection prevention and control**	Not specified
**… - Training /education (incl. in- outdoor training)**	Not specified
**… - Staff hygiene (incl. PPE, Hand hygiene)**	Not specified
**… - Relative/visitors (incl. Physicians/Therapists)**	Not specified
**… - Cleaning and disinfection aspects**	Cleaning Methods<br>A: microbial reduction, but poor reliability + reproducibility

	B: complete microbial elimination in all tested samples<br>C: reduced microbial burden, if secure fixation in a vertical position in dishwasher<br>D: did not improve results of manual pre-cleaning<br><br>*(more details: s. Tab. 2)*
**… - Handling of medical devices**	*s. cleaning and disinfection aspects*
**… - Waste management**	Not specified
**... - Infectious critical activities (incl. MRSA, Screening, Surveillance)**	Not specified
**… - Caring for persons (prevention and infection control)**	Not specified
**… - Handling of medication**	Not specified
**… - Laundry hygiene**	Not specified
**… - Kitchen hygiene**	Not specified
**Conclusion/Limitations**	

Conclusion:
- No standardized cleaning procedures or management policies on use + reuse of tracheostomy tubes exist
- Inner lumen of tracheostomy tubes is colonised by Staph. aureus, Staph. epidermis, Pseudomonas aeruginosa + Candida spp. most frequently
- Recommendation: manual cleaning + chemical or thermal disinfection (dishwasher seems to be optimal method for regular cleansing + disinfecting tracheostomy tubes, if tubes are placed in secure fixation)
- No signs of material alteration
- Cuff functionality after the single reprocessing regiments remained intact in all but one cuffed tubes

Limitations:
- No investigation on microbial reduction which can be achieved by using a dishwasher alone
- Only one single reprocessing step

Notes for review:
- Some aspects remain unclear (e.g. setting + country)
- No limitations discussed

Abbreviations:

US = ultrasound

Appendix 3: Data Charting Sheets. Hygiene Management for Long-term Ventilated Persons in the Home Health Care Setting: A Scoping Review

Data charting sheet - for health care professional, patients, relatives	
**Study description**	
**First author**	Neumann, Nadja
**Title**	Multidrug-resistant organisms (MDRO) in patients in outpatient care in Rhine-Mine region, Germany, in 2014. Prevalence and risk factors
**Year**	2015
**Country**	Germany
**Study aims**	- Prevalence of MDRO<br>- Identification of risk factors for MDRO<br>- Finding differences in IC nursing services vs. other nursing services with regard to the colonisation of MRSA/ MDRO
**Methodology/Measures**	**Study design:** observational/ descriptive study<br><br>**Period data collection:** Oct – Dec 2014<br><br>**Recruitment:** 40/nursing services in Rhein-Main area were asked to take part (10/40 agreed to participate)<br><br>**Measures:**<br>a. Anamnesis<br>- HALT-questionnaire: survey instrument to investigate infections + antibiotic use in nursing homes in Europe<br><br>b. Swabs (nose, throat, anal)<br>- Laboratory analysis of swabs (MRSA + MDRO)<br><br>**Statistical Analysis:**<br>- Differences nursing services with IC vs. without IC (Mann-Whitney, Odds Ratios, regression)
**Setting, Participants**	
**Sample size**	486 patients (=10 nursing services in Rhine-Main area)<br>- **All patients** anamnestic survey<br>- **269 (55,3%)** throat and/or nasal swab<br>- **132 (27,3%)** anal swab
**Sex**	32,9% of all patients male sex (50% male sex in IC group)
**Age of participation**	36,9% (0% IC) >85 years
**Kind of disease**	Significant differences between nursing services:

	Nursing services without IC / Nursing services with IC:<br>Incontinence: 27% / 70%<br>Disorientation: 20% / 40%<br>Level of care dependency >3: 11% / 55%<br>Wheelchair assisted: 16% / 55%<br>Urinary catheter: 6% / 55%<br>Tracheostomy: 0% / 75%<br>Invasive ventilation: 0% / 70%<br>PEG-Sonde: 2% / 70%<br><br>*(more detail: Tab. 1)*
**Kind of artificial ventilation (incl. duration)**	No patient without IC had tracheostomy vs. 75% (15/20) patients of IC<br>- Invasively ventilated: 70% (14/20) of IC-patients
**Family participation**	Not specified
**Concept of home-based setting/Licensed beds**	10 nursing services in Rhine-Main area<br>- 9 “normal” nursing service<br>- 1 IC service
**Professions/Qualifications involved/Duration professional employment**	Ambulatory nursing services (normal + intensive)
**Cooperation (e.g. Lung specialist, Weaning centre)**	Not specified
**Key Findings - Hygiene management**	
**Quality management for hygiene, e. g. Infection control practitioner, duration h for infection prevention and control**	Not specified
**… - Training /education (incl. in- outdoor training)**	Not specified
**… - Staff hygiene (incl. PPE, Hand hygiene)**	Not specified
**… - Relative/visitors (incl. Physicians/Therapists)**	Not specified
**… - Cleaning and disinfection aspects**	In case of MRSA-detection, requesting a free sanitation kit was possible
**… - Handling of medical devices**	Not specified
**… - Waste management**	Not specified
**... - Infectious critical activities (incl. MRSA, Screening, Surveillance)**	**Colonisation** / **Significant risk factors**:<br>MRSA: - Level of care dependency >3; - Hospitalisation < 6 months<br>ESBL: - Disorientation; - Hospitalisation < 6 months

Nested table (first row):

	Nursing services without IC	Nursing services with IC
Incontinence	27%	70%
Disorientation	20%	40%
Level of care dependency >3	11%	55%
Wheelchair assisted	16%	55%
Urinary catheter	6%	55%
Tracheostomy	0%	75%
Invasive ventilation	0%	70%
PEG-Sonde	2%	70%

	MDRO: - Catheter/ stomata<br>- Level of care dependency >3<br>- Positive history for MRSA
**… - Caring for persons (prevention and infection control)**	Not specified
**… - Handling of medication**	1% of patients received an antibiotic on the day the smear was taken
**… - Laundry hygiene**	Not specified
**… - Kitchen hygiene**	Not specified
**Microbiological findings**	

Significant differences between nursing services:

	"normal" nursing services	IC-nursing services	All services
MRSA colonisation	12.5%	3.2%	3.7%
3MRGN and/or ESBL	13.1%	30%	14.4.%
3MRGN	30%	5.7	7.6%
4MRGN	0	0	0

Conclusion/Limitations

Conclusion:

- **High prevalence of MRSA + MDRO-colonisation in outpatient care**
  → 3MRGN-colonisation 3x as high as in normal population
  → essential: good hygiene procedures (hand hygiene, wound management, punctures/ injections, catheters, stomata, artificial respiratory)
  → hospital hygiene + infection prevention to be applied in outpatient care (KRINKO)
- **Significant risk factors for MRSA/ MDRO colonisation:** hospitalisation < 6 months, level of care dependency > 3, disorientation, catheter/ stomata, positive history for MRSA

Notes for ScR:

- No limitations discussed

Abbreviations:

ESBL = Erreger mit erweiterter Resistenz gegen Beta-Laktamantibiotika

HALT = healthcare-associated infections in long-term care facilities

IC = intensive care

MDRO = MRGN/ MRE = multiresistente (gramnegative) Erreger (3MRGN/ 4MRGN = Resistenz gegen Leitantibiotika aus drei/vier Antibiotikagruppen)

Appendix 3: Data Charting Sheets. Hygiene Management for Long-term Ventilated Persons in the Home Health Care Setting: A Scoping Review

**Data charting sheet - for health care professional, patients, relatives**	
**Study description**	
**First author**	Prasad, Nishant
**Title**	Asymptomatic rectal colonization with carbapenem-resistant *Enterobacteriaceae* and *Clostridium difficile* among residents of a long-term care facility in New York City
**Year**	2016
**Country**	USA
**Study aims**	To determine prevalence of asymptomatic rectal colonisation with CDI or CRE in LTCF
**Methodology/Measures**	**Study design:** cross-sectional study<br><br>**Method:**<br>Rectal swabs over a 17-week period<br>- Molecular analysis in laboratory<br>- Analysis of risk factors or CRE + CDI<br><br>**Exclusion criteria:**<br>- Signs + symptoms of diarrhea<br>- Documented CDI-associated infection/ treatment of CDI-infection<br>- Residents on short-term rehabilitation floor<br><br>**Data analysis:**<br>- Statistical analysis using SAS<br>- Independent t-test, fisher exact-test, logistic regression, p-values
**Setting, Participants**	
**Sample size**	301 patients
**Sex**	63% female
**Age of participation**	75 years (median age); only adult patients
**Kind of disease**	Comorbid conditions:<br>- 84% hypertension<br>- 56% hyperlipidemia<br>- 49% diabetes mellitus<br>- 43% dementia<br>- 37% coronary heart disease<br>- 34% congestive heart failure<br>- 26% COPD
**Kind of artificial ventilation (incl. duration)**	Ventilated patients:

	- Represented 41% of cohort<br>- Over 90% external feeding<br>- >30% had prior CDI-infection<br>- 53% PPI within the last 60 days
**Family participation**	Not specified
**Concept of home-based setting/Licensed beds**	80 bed ventilator unit (in a 320-bed medical centre-affiliated LTCF)
**Professions/Qualifications involved/Duration professional employment**	Not specified
**Cooperation (e. g. Lung specialist, Weaning centre)**	Not specified
**Key Findings - Hygiene management**	
**Quality management for hygiene, e. g. Infection control practitioner, duration h for infection prevention and control**	Infection control isolation policies (not further described)
**… - Training /education (incl. in- outdoor training)**	Not specified
**… - Staff hygiene (incl. PPE, Hand hygiene)**	Not specified
**… - Relative/visitors (incl. Physicians/Therapists)**	Not specified
**… - Cleaning and disinfection aspects**	Not specified
**… - Handling of medical devices**	Not specified
**… - Waste management**	Not specified
**… - Infectious critical activities (incl. MRSA, Screening, Surveillance)**	*S. microbiological findings above*
**… - Caring for persons (prevention and infection control)**	Not specified
**… - Handling of medication**	Not specified
**… - Laundry hygiene**	Not specified
**… - Kitchen hygiene**	Not specified
**Microbiological findings**	

**Colonisation rates**	- 19% asymptomatic CDI-colonisation<br>- 19% CRE-colonisation
**Colonisation with CRE was associated with**	- 19% history of CDI -infection (median 240 days since prior episode)<br>- 45% history of CDI -infection + asymptomatic colonisation with CDI<br>- 26% history of CDI -infection + colonisation of CRE<br>- 17% history of CDI -infection + colonisation with CRE + CDI
**Factors associated with CDI, CRE + concurrent CDI/ CRE**	- Tracheostomy collar<br>- Location on respiratory floor<br>- Colonisation with CRE

	- Colonisation with CRE was associated with: MV, enteral feeds, PPI in the last 60 days, location on respiratory floors, hyperlipidaemia, COPD, colonisation with CDI<br>- for patients with prior CDI-infection: lower number of days since prior CDI-infection was significantly associated with an increased risk of CDI-colonisation + concurrent CDI/CRE-colonisation

Conclusion/Limitations	

Conclusion:
- strong association of colonisation with CDI/ CRE with disruption of normal flora (by MV/ enteral feeds) + prior CDI-infection

Limitations:
- retrospective analysis
- multiple persons collected swabs + tabulated data
- study was conducted in a single LTCF → results may not be generalizable to others with different characteristics
- no data on antibiotic use available

Notes for review:
- Setting: stationary hospital, not outpatient care
- No hygiene management

Abbreviations:

CDI = Clostridium difficile

CRE = carbapenem-resistant Enterobacteriacae

LTCF = long-term care facility

MV = mechanical ventilation

PPI = proton pump inhibitor

Data charting sheet - for health care professional, patients, relatives	
**Study description**	
**First author**	Schwerdtner, Nora-Lynn
**Title**	Herausforderungen im Umgang mit MRE in außerklinischen Intensivpflege-Wohngemeinschaften Erfahrungsbericht und Ergebnisse einer Prävalenzerhebung zu multiresistenten Erregern im Stadtgebiet Jena
**Year**	2020
**Country**	Germany
**Study aims**	Prevalence of MDRO + evaluation of hygiene management in AKI-flat-sharing community in Jena
**Methodology/Measures**	**Study design:** observational/ descriptive<br><br>**Background:** Reported evidence of MDRO-infection for initially 3 residents in 1 FSC<br><br>→ Occasional inspection + prevalence survey (MDRO-screening) of affected FSC<br>→ Additional survey of 3 providers of IC-FSC<br><br>**Data Collection:** (see table below)<br><br>**Data Analysis:** Descriptive

Data Collection:

Occasional, unannounced inspection + prevalence survey	MDRO-prevalence survey
In affected FSC	In all 3 FSC
- Screening for MDRO + MRSA: nose/throat, groin, rectal swabs + on devices<br>- Inspection: Evaluation of practiced hygiene management	- standardized questionnaires for structural data (number + qualification of med. staff, number of clients cared for, number of beds, type + number of devices)<br>- Anamnestic data: MDRO colonisation rates
Repeated screening after implementation of hygiene management requirements	

Data Analysis: Descriptive

Appendix 3: Data Charting Sheets. Hygiene Management for Long-term Ventilated Persons in the Home Health Care Setting: A Scoping Review

**Setting, Participants**	
**Sample size**	3/4 known FSC in Jena<br>24 patients
**Sex**	Not specified
**Age of participation**	3 patients < 18 years old
**Kind of disease**	- **18/24** (75%) level of care dependency 4 + 5<br>- **4/24** (17%) mobile on their own<br><br>In total 75 devices:<br>- **21/24** (88%) PEG-Sonde<br>- **11/24** (46%) urinary catheter<br>- **4/24** (17%) suprapubic catheter<br>- **7/24** (29%) wounds
**Kind of artificial ventilation (incl. duration)**	- **4/24** (17%) permanent respirator<br>- **7/24** (29%) <24h respirator<br>- **21/24** (88%) Tracheal cannula
**Family participation**	Not specified
**Concept of home-based setting/Licensed beds**	- 3 FSC (supervised by 2/3 nursing services in Jena)<br>- 24/40 (60%) occupied beds
**Professions/Qualifications involved/Duration professional employment**	In total 60 employees in 3 FSC<br>- 14 geriatric nurses (Altenpfleger)<br>- 17 certified nurse (Krankenpfleger)<br>- 3 nursing assistant (Pflegehelfer)<br>- 4 other
**Cooperation (e. g. Lung specialist, Weaning centre)**	Not specified
**Key Findings - Hygiene management**	
**Quality management for hygiene, e. g. Infection control practitioner, duration h for infection prevention and control**	Occasional inspection in affected FSC:<br>- Desolate hygiene management (e.g. hygiene plan not available, incorrect/ missing information on disinfection, contamination on surfaces + medical equipment)<br>- Insufficient structural + technical requirements
**… - Training /education (incl. in- outdoor training)**	Occasional inspection: knowledge deficits regarding basic hygiene despite annual training
**… - Staff hygiene (incl. PPE, Hand hygiene)**	1/3 FSC: missing hygiene equipment such as hand disinfectant dispensers and washbasins in nursing staff' dressing rooms
**… - Relative/visitors (incl. Physicians/Therapists)**	Not specified
**… - Cleaning and disinfection aspects**	Not specified

**… - Handling of medical devices**	Not specified
**… - Waste management**	Not specified
**… - Infectious critical activities (incl. MRSA, Screening, Surveillance)**	- **1/3 FSC** admission screening by GP
**… - Caring for persons (prevention and infection control)**	Not specified
**… - Handling of medication**	- **18/24** (75%) patients received antibiotics < 6 months
**… - Laundry hygiene**	Not specified
**… - Kitchen hygiene**	Not specified
**Microbiological findings**	

**Anamnestic MRE evidence in 3 WG**	**Occasional Screening in 1 AKI**
- 54% positive MRE-evidence<br>- 29% 3MRGN<br>- 23% 4MRGN<br>- 21% MRSA	- 6/6 patients with multiple colonisation (14 positive MDRO- evidences)<br>- 3MRGN + 4MRGN were found both on medical devices (tracheal cannulae, chatheter + PEG-Sonde) + patients themselfes<br>- MRSA detection on tracheal cannulae<br><br>*(for more details s. p. 7, Tab. 2)*

Conclusion/Limitations

Conclusion:
- Multiple colonisations in 1 FSC is attributed to desolate hygiene management
- High device application rate in all 3 FSC (75 devices for 24 patients)

Limitation:
- Anamnestic survey was verified by screening only in 1 FSC
- City of Jena only: small database

Notes for Review:
- Desolate hygiene management only described as an example with experts from the inspection report
- Hygiene management of other 2 FSC not described at all

Abbreviations:

AKI = außerklinische Intensivpflege
MDRO = multidrug resistant organisms (MRE, MRGN)
FSC = flat-sharing community
GP = general practitioner

<table>
<tr><th colspan="2">Data charting sheet - for health care professional, patients, relatives</th></tr>
<tr><th>Study description</th><th></th></tr>
<tr><td>First author</td><td>Toussaint, Michel</td></tr>
<tr><td>Title</td><td>Is disinfection of mechanical ventilation tubing needed at home?</td></tr>
<tr><td>Year</td><td>2006</td></tr>
<tr><td>Country</td><td>Belgium</td></tr>
<tr><td>Study aims</td><td>- To determine the HVC cleanliness + sterility of HVC used by home ventilated patients
- To determine efficiency of tubing cleaning + decontamination protocols recommended to patients</td></tr>
<tr><td>Methodology/Measures</td><td>Study design: Prospective Cohort Study

Method:
- Prior to the study: Patients had received “Low Level Decontamination + Cleaning”-Protocol (written + taught); used for > 12 months (s. category “Training”)
- 39 used + 7 new EVAs (as control) were examined in 2 different experiments:

<table>
<tr><th></th><th>Experiment 1</th><th>Experiment 2</th></tr>
<tr><td>Where</td><td colspan="2">During hospitalization OR during patient consultations in the centre for MV</td></tr>
<tr><td>When</td><td>After the patient arrived</td><td>- The day after Exp. 1
- After cleaning done by investigators</td></tr>
<tr><td>Who</td><td colspan="2">Exp. 1 + 2 by the same investigator</td></tr>
<tr><td>What</td><td>Analysis:

1. Visual (EVA + complete circuit):
- 10-point scale (judged “dirty” = 2/10 or lower) + 4 criteria (*) of severity of visual dirtiness
- 10 points maximum was also given for the full circuit with averaging of the mean score of the individual tubing parts

(*) colour (1 point = light; 3 points = dark); (2) incrustation (0 = disappears; 2 = stays); (3) extent (1 = particle; 2 =</td><td>a. Cleaning by investigators (decided at random):
- Sequence A: HAC-cleaning (decontamination by 15min submersion in a chemical 5% HAC-bath)
- Sequence B: dishwasher (90min programme at a temperature >65°C + classical dishwasher powder)

b. Analysis
- s. Exp. 1</td></tr>
</table>
</td></tr>
</table>

		surface > 1 qcm); (4) relative humidity (0 = dry; 3 0 = humid)<br><br>**2. Bacteriological** (EVA only):<br>dry swab on the EVA balloon + evaluation in laboratory	
	Statistics	Exp. 1 + 2 results for visual + bacteriological analysis were compared (paired + unpaired t-tests; correlations), p value 0.01	

**Setting, Participants**	
**Sample size**	39 severe restrictive ventilated patients
**Sex**	Not specified
**Age of participation**	Not specified
**Kind of disease**	- **All** patients: functional tetraplegy with chronic alveolar hypoventilation<br>- **24/39** Duchenne muscular dystrophies<br>- **4/39** congenital myopathies<br>- **4/39** spinal muscular atrophies<br>- **4/39** tetraplegics<br>- **2/39** polyneuropathies<br>- **1/39** limb girdle dystrophy
**Kind of artificial ventilation (incl. duration)**	- **All** respirators volumetric<br>- **All** patients ventilated at home with EVA for >12 months (mean time ventilated: 7.7 years)<br>- **All** patients: ventilation + tubing with valve between 6-12 months<br>- **Group (T): 16/39** tracheostomy (circuits connected to humidifier + water trap)<br>- **Group (N): 23/39** noninvasively with nasal mask (13 silicone custom made mask; 10 commercial mask)<br>- **24/39** continuously ventilated (16 by tracheostomy; 8 by mouthpiece during the day/ nasal mask at night)
**Family participation**	Not specified
**Concept of home-based setting/Licensed beds**	- **11/39** lived in institution<br>- **28/39** lived at home
**Professions/Qualifications involved/Duration professional employment**	Not specified
**Cooperation (e. g. Lung specialist, Weaning centre)**	Not specified
**Key Findings - Hygiene management**	

**Quality management for hygiene, e. g. Infection control practitioner, duration h for infection prevention and control**	Not specified
**… - Training /education (incl. in- outdoor training)**	**Patients**:<br>- Maintenance advice for HVC (*) is not well adhered to (even taught + written)<br>- Need to be taught in hospital + repetition at regular intervals with short trainings<br>- 10-point scale could be used to send feedbacks to patients<br><br>(*) "Low-Level Decontamination + Cleaning"-Protocol:<br>- To clean macroscopic spots with detergent + hot water followed by a 15min HAC-bath (diluted to 5%)<br>- 2x per month: circuit, tubing + interfaces decontamination by HAC<br>- To clean cannulae of tracheostomy with hydrogen peroxides (oxygen water)<br><br>**Professionals**:<br>- Clearly need additional training sessions to improve their expertise in respiratory equipment
**… - Staff hygiene (incl. PPE, Hand hygiene)**	Not specified
**… - Relative/visitors (incl. Physicians/Therapists)**	Not specified
**… - Cleaning and disinfection aspects**	2 different cleanings are described in the study:<br>- "Low-Level Decontamination + Cleaning"-Protocol *(s. category "training" above)*<br>- Cleaning by investigator during Exp. 2 *(s. category "methodology" above)*<br><br>ICU-hygiene protocols are not appropriate for home use, because they only recommend inadequate disinfection + fail in advising simple washing<br>→ need for maintenance protocols aiming for cleanliness, not for sterility<br>→ **Recommendation by authors**: low-level-disinfection in dishwasher<br>- All pieces of the circuit (masks, EVAs…) at 70°C + 90min<br>- Available in most families + institutions<br>- Easy of use
**… - Handling of medical devices**	*(s. "cleaning + disinfection aspects" above)*
**… - Waste management**	Not specified
**... - Infectious critical activities (incl. MRSA, Screening, Surveillance)**	Not specified
**… - Caring for persons (prevention and infection control)**	Not specified
**… - Handling of medication**	Not specified
**… - Laundry hygiene**	Not specified
**… - Kitchen hygiene**	Not specified

**Microbiological findings**	
**Experiment 1:**<br>- Dirtiness was worse in (T) than in (N)<br>- EVA in (T) were more contamined than in (N)<br>- Contamination rates (N): 22% (no presence of PPO)<br>- Contamination rate (T): 81% (19% were PPO)<br>- Significant correlation was found between the dirtiness of the complete tube + EVA<br>- 69% of EVA + 56% of circuits were visually dirty<br>- Circuits from patients living at home were visually cleaner than those from patients living in institutions<br>- 46% of the valves were contamined by microorganisms + 22% by fungi (seldom contamined by potentially pathogenic organisms)<br><br>**Experiment 2:**<br>- Remained dirty after dishwasher cleaning (HAC bath): 14% (64%) of dirty EVA → EVA cleanliness was significantly better after dishwasher cleaning with similar bacteriological decontamination<br>- choice of the dishwasher did not play any role in the quality of cleaning because all recent machines are equivalent	
**Conclusion/Limitations**	
**Conclusion:**<br>- Circuits from patients living at home were visually cleaner than those from patients living in institutions → nurses in institutions need additional training sessions to improve their expertise in respirator equipment maintenance<br>- Maintenance advice for HVC is not well adhered to → patients + their families need trainings in hospital to demonstrate the utility of simple basic hygiene<br>- Dishwasher cleaning is more efficient than chemical HAC-bath → low-level disinfection is efficient + possible for families, patients + institutions<br><br>**Notes for Review:**<br>- There are no limitations discussed!!<br>- Method of visual assessment remains unclear (10 point scale + 4 criteria of severity) + why < 2/10 decelerated as "dirty"?	

Abbreviations:

EVA = expiratory valves
HAC = Hospital Antiseptic Concentrate
HVC = home ventilation circuits
ICU = intensive care unit
MV = mechanical ventilation
